# Molecular dynamics simulations data of six compounds F3J-BRD4/CBP, EX1-BRD4/CBP, and E2T-BRD4/CBP

**DOI:** 10.1016/j.dib.2021.107009

**Published:** 2021-03-27

**Authors:** Shiliang Wu, Lifei Wang, Lulu Zhang, Xiaoyan Xu, Juan Zhao

**Affiliations:** School of Science, Shandong Jiaotong University, Jinan 250357, China

**Keywords:** Bromodomain-containing protein 4, CREB binding protein, Molecular dynamics simulations, Principal component analysis, Residue-based free energy decomposition

## Abstract

The data here described are related to the research article entitled “Molecular dynamics insights into binding selectivity of inhibitors toward BRD4 and CBP” [1]. Bromodomain-containing protein 4 (BRD4) and CREB binding protein (CBP) play important roles in tumorigenesis and development. We performed 200-ns molecular dynamics (MD) simulations on three pairs of inhibitor-BRD4 and inhibitor-CBP complexes to clarify binding selectivity of inhibitors toward BRD4 and CBP. Principal component (PC) analysis was used to probe changes in internal dynamics and conformations of BRD4 and CBP due to inhibitor bindings. Analysis of residue-based free energy decomposition was employed to explore the roles of separate residues in binding selectivity of inhibitors to BRD4 versus CBP.

## Specifications Table

**Table:** 

Subject	Biology, Physics
Specific subject area	Computational Molecular Biophysics, Molecular Dynamics Simulations
Type of data	Table, figure
How data were acquired	Preparation of systems with PyMOL software, VMD, and ambertools, molecular dynamics simulations with Amber18
Data format	Analyzed, raw
Parameters for data collection	The force field parameters of inhibitors, proteins, and water molecules for MD simulations separately stem from the General Amber force field (GAFF), the Amber ff14SB force field and TIP3P model. MD simulations was operated at 300 K and 1 atm.
Description of data collection	MD simulations were performed using Amber18 to generate structural ensembles, PC analysis was conducted with the module CPPTRAJ in Amber 18.
Data source location	Institution: School of Science, Shandong Jiaotong University City/Town/Region: Jinan Country: China
Data accessibility	Data is stored in a public repository. Repository name: Mendeley Data Data identification number: http://dx.doi.org/10.17632/5vvmdbtcd5.5 Direct URL to data: https://data.mendeley.com/datasets/5vvmdbtcd5/5
Related research article	Shiliang Wu, Lifei Wang, Lulu Zhang, Xiaoyan Xu, Juan Zhao, Molecular dynamics insights into binding selectivity of inhibitors toward BRD4 and CBP, Chem. Phys. Lett. 769 (2021) 138435. https://doi.org/10.1016/j.cplett.2021.138435

## Value of the Data

• These data are useful for understanding internal dynamics and conformations of BRD4 and CBP due to inhibitor bindings.
• Researchers in the field of biology, physics and drug design can benefit from these data in exploring the efficient targets of dual inhibitors toward BRD4 and CBP.
• These data might be used for further insights into binding selectivity of inhibitors toward BRD4 and CBP.

## 1 Data Description

**Fig. 1:**
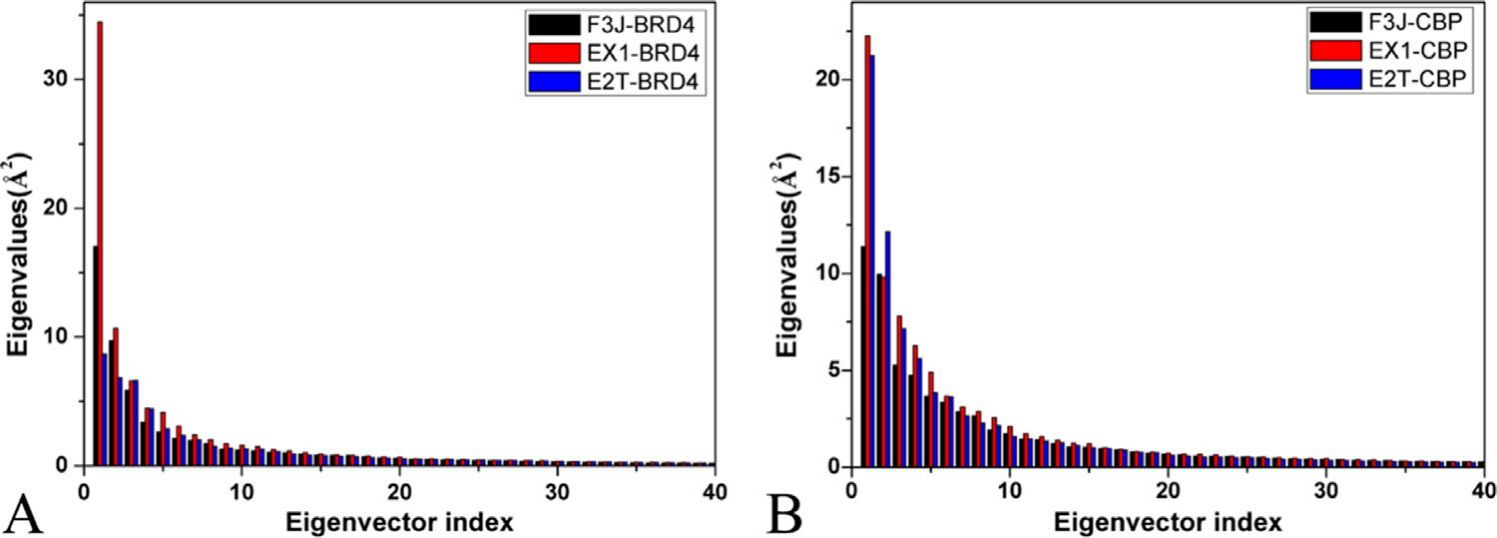
The function of the eigenvalues versus the eigenvector index stemming from the diagnolization of the C_α_ atoms covariance matrix in the BRD4 and CBP complexed with three inhibitors F3J, EX1 and E2T.

**Fig. 2:**
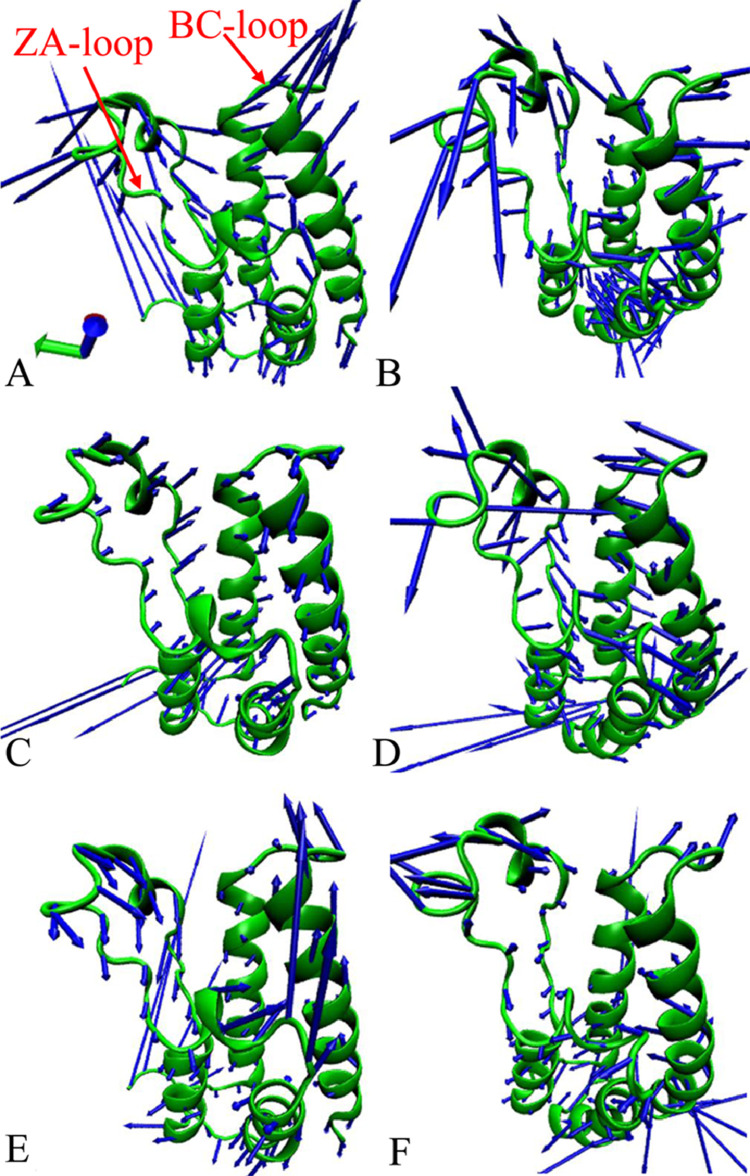
Collective motions of BRD4 and CBP corresponding to the first eigenvector from PC analysis on the equilibrated MD trajectories: (A) the F3J/BRD4 compound, (B) the F3J/CBP compound, (C) the EX1/BRD4 compound, (D) the EX1/CBP compound, (E) the E2T/BRD4 compound, and (F) the E2T/CBP compound. In this figure the direction of the arrow is an indicator of the collective motion direction, and the length of the arrow represents the strength of the movements.

**Fig. 3:**
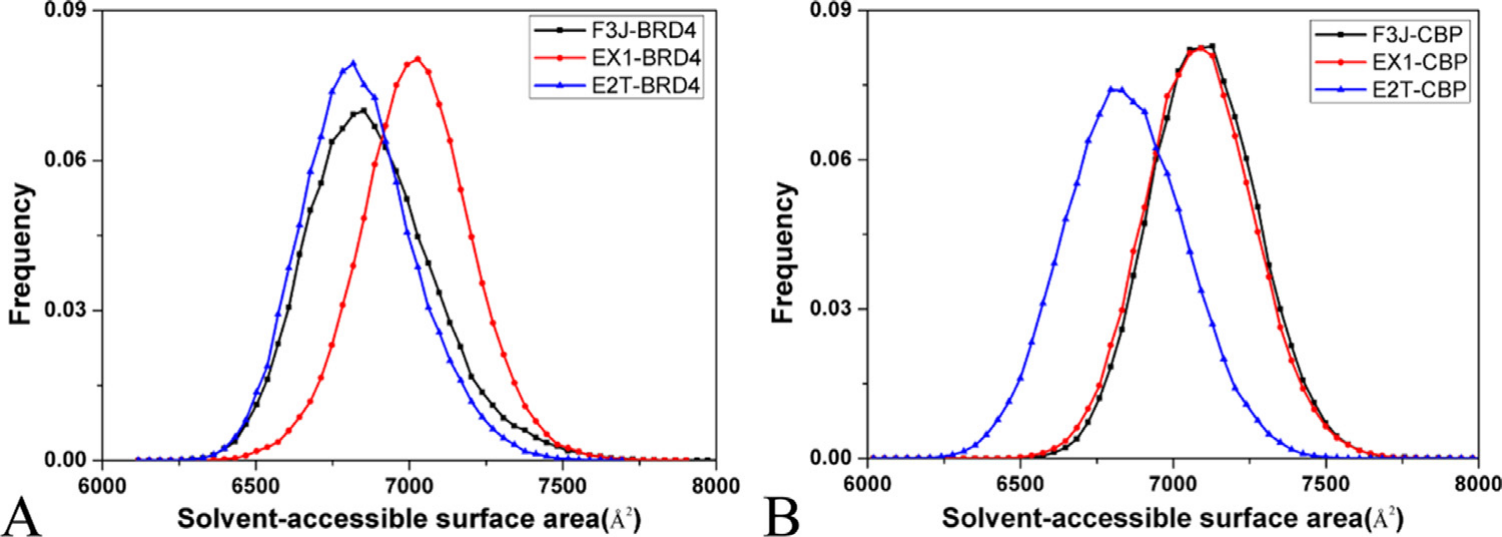
Frequency distributions of molecular surface area (MSA) for the inhibitor-BRD4/CBP complexes, in which the *x*-axis shows the MSA and the *y*-axis indicates the frequency distributions of the MSA.

**Fig. 4:**
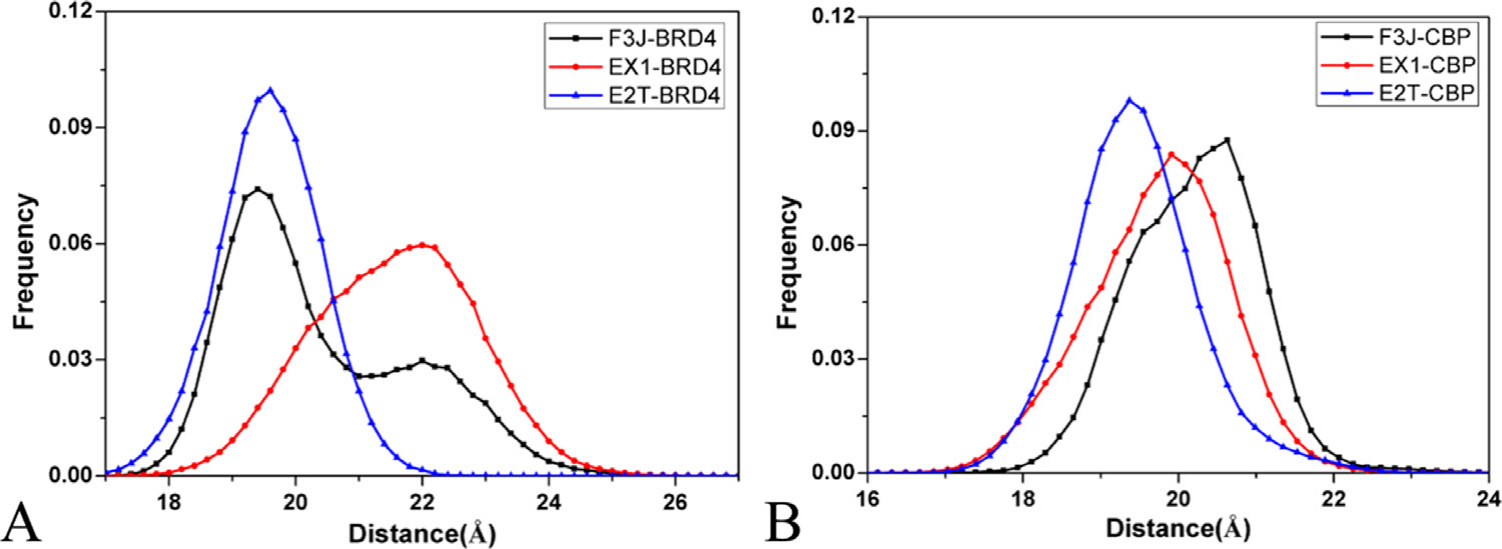
(A) Frequency distributions of distance between Leu92 (in the ZA loop of BRD4) and Pro142 (in the BC loop of BRD4), (B) frequency distributions of distance between Leu1120 (in the ZA loop of CBP) and Lys1170 (in the BC loop of CBP).

The data described here are derived from insights into binding selectivity of inhibitors toward BRD4 and CBP by using MD simulations. The analyses were performed on six systems, including the F3J-BRD4/CBP, EX1-BRD4/CBP, and E2T-BRD4/CBP complexes. Fig. 1 exhibits the plots of the eigenvalues against eigenvector indexes obtained from the diagonalization of the covariance matrix constructed by utilizing atomic coordinates saved at MD trajectories. Six porcupine plots are depicted in Fig. 2 by employing the first eigenvector, the VMD software [2], k.txt file, and the initialized structure. Molecular surface area (MSA) of six systems were calculated by the CPPTRAJ module in AMBER18, and the frequency distributions of MSA are indicated in Fig. 3. We measured the distance between the C_α_ atoms of Leu92/Leu1120 in the ZA-loop of BRD4/CBP and that of Pro142/Lys1170 in the BC-loop of BRD4/CBP, representing the distance of the ZA-loop away from the BC-loop and their frequency distributions are showed in Fig. 4. The raw data for four figures have been uploaded to Mendeley Data repository: Eigenvalue for Fig. 1, Porcupine for Fig. 2, MSA for Fig. 3, and Loop for Fig. 4. The Porcupine folder in Mendeley Data shows the first six eigenvectors (fort.71–fort.76), and the initialized structure of six complexes, and k.txt file. Take fort.71 file as an example, the first triple of numbers determine coordinates of the Cα atoms of the residues 53–166 in BRD4 and that of the residues 1083–1196 in CBP. They defined the starting point, and the unit is Å. The second triple of numbers show the motion directions of BRD4 and CBP, and they represent the movement vectors. The MSA folder includes the data of Molecular surface area (MSA) of six systems, each .dat file gives the MSA of 100,000 conformations, the number in the second column represents MSA of each conformation and the unit is Å^2^. To calculate relative frequency from the raw data for each compound, 55 intervals were derived from 6000 to 8000 Å^2^ at an increment of 37 Å^2^ and the results were depicted in Fig. 3. The Loop folder indicates the data of the distance between the Cα atoms of Leu92/Leu1120 in the ZA-loop of BRD4/CBP and that of Pro142/Lys1170 in the BC-loop of BRD4/CBP, representing the distance of the ZA-loop away from the BC-loop, each .dat file gives the distance of 100,000 conformations, the number in the second column represents distance of the ZA-loop away from the BC-loop in each conformation and the unit is Å. To calculate relative frequency from the raw data for each compound, 50 intervals were derived from 15.5 to 24.5 Å at an increment of 0.18 Å and the results were depicted in Fig. 4. Tables 1 and 2 elucidates the contributions of per-residue to binding free energies from backbone and sidechains of residues.

**Table 1:** Decomposition of free energies on a per-residue basis of inhibitor-BRD4 compounds^a^.

	F3J-BRD4			EX1-BRD4			E2T-BRD4		
	bS_GBTOT_	bB_GBTOT_	bT_GBTOT_	S_GBTOT_	B_GBTOT_	T_GBTOT_	S_GBTOT_	B_GBTOT_	T_GBTOT_
Trp81	−2.10	−0.28	−2.38	−2.63	−0.36	−3.00	−0.62	−0.16	−0.77
Pro82	−1.68	0.07	−1.62	−1.62	0.03	−1.60	−1.64	−0.47	−2.11
Gln85	−0.46	0.06	−0.39	−1.39	0.03	−1.36	−0.50	−0.02	−0.53
Val87	−1.82	−0.42	−2.25	−2.02	−0.39	−2.44	−1.90	−1.22	−3.12
Leu92	−1.55	0.00	−1.55	−0.84	0.00	−0.85	−2.50	−0.03	−2.54
Leu94	−0.57	−0.01	−0.59	−0.47	0.01	−0.47	−1.03	0.00	−1.03
Asn140	−1.62	0.02	−1.60	−0.88	0.04	−0.85	−0.80	0.01	−0.80
Ile146	−1.99	−0.12	−2.12	−1.50	−0.06	−1.57	−1.86	−0.06	−1.93

a All values are in kcal/mol.

b The contributions of sidechain, backbone and total to the inhibitor-residue interactions.

**Table 2:** Decomposition of free energies on a per-residue basis of inhibitor-CBP compounds^a^.

	F3J-CBP			EX1-CBP			E2T-CBP		
	bS_GBTOT_	bB_GBTOT_	bT_GBTOT_	S_GBTOT_	B_GBTOT_	T_GBTOT_	S_GBTOT_	B_GBTOT_	T_GBTOT_
Leu1109	−0.88	−0.22	−1.10	−0.84	−0.22	−1.06	−1.47	−0.46	−1.92
Pro1110	−1.59	−0.32	−1.91	−1.75	−0.16	−1.92	−2.22	−0.29	−2.51
Gln1113	−1.10	−0.02	−1.11	−0.53	0.02	−0.51	−0.55	0.08	−0.48
Val1115	−1.34	−0.32	−1.66	−1.20	−0.16	−1.37	−1.19	−0.25	−1.45
Leu1120	−1.93	0.16	−1.79	−2.75	−0.13	−2.89	−2.41	0.00	−2.41
Ile1122	−3.25	−0.36	−3.63	−0.57	0.00	−0.56	−1.22	−0.01	−1.23
Asn1168	−0.58	0.03	−0.54	−1.47	0.02	−1.45	−1.42	0.03	−1.39
Val1174	−1.65	−0.09	−1.75	−1.24	−0.07	−1.31	−1.81	−0.11	−1.92

a All values are in kcal/mol.

b The contributions of sidechain, backbone and total to the inhibitor-residue interactions.

## 2 Experimental Design, Materials and Methods

### 2.1 Preparation of systems

Three crystal structures were taken from the Protein Data Bank (PDB): F3J-BRD4 for 6CIY, EX1-BRD4 for 6CD4 [3], and E2T-CBP for 6FQO [4]. Because the crystal structures of E2T-BRD4, F3J-CBP, and EX1-CBP complexes are unavailable in PDB, the inhibitors E2T, F3J, and EX1 were put into binding site of BRD4 and CBP by using the PyMOL software and structural superimposition. BRD4 and CBP correlating with PDB ID 6CJ1 (BRD4) [3] and 6FQO (CBP) are used as templates to perform structural superimposition. The lacking hydrogen atoms in crystal structures were added to the interrelated heavy atoms with the help of the Leap module in AMBER18 [5]. The protonation status of residues in BRD4 and CBP were examined by using PROPKA program [6]. Bond-charge correction (BCC) calculations were carried out employing the AM1 method in Antechamber module , and then the charges were assigned to separate atoms of inhibitors. The force field parameters of proteins, inhibitors, and water molecules for MD simulations separately stem from the Amber ff14SB force field [7], the General Amber force field (GAFF) [8], and TIP3P model. Then four Cl- and three Na+ were separately added around the inhibitor-BRD4 compounds and inhibitor-CBP complexes to neutralize the systems.

### 2.2 Molecular dynamics simulations

Energy minimizations and MD simulations were performed by using the PMEMD module in AMBER18 [9]. To eliminate any unfavorable factors because of the system initialization, a two-stage energy optimization was carried out for each system: (1) to minimize water molecules and ions by constraining the complexes; (2) to optimize the whole system without any restrictions. During optimization, each system was subjected to the steepest descent minimization of 2500 steps and the conjugate gradient minimization of 2500 steps. Each system was heated from 0 to 300 K in 2 ns, and then equilibrated for 1 ns at a temperature of 300 K. The Langevin thermostat [10] and the Particle Mesh Ewald (PME) method  were used to control the temperature and calculate long-range electrostatic interactions of each system, respectively. With the help of the SHAKE algorithm [11], the expansion and contraction of all covalent bonds linking to hydrogen atoms was restricted. Electrostatics interactions and van der Waals interactions between atoms were truncated at a rational cutoff value of 10 Å. Finally, 200-ns MD simulation for each system without any restrictions was operated at 300 K and 1 atm to relax each systems and generate structural ensembles for post-processing analysis [12].

### 2.3 Principal component analysis

In this work, PC analysis [13], were carried out by using the CPPTRAJ module in Amber18 [14]. Firstly a covariance matrix C should be constructed by means of the coordinates of the C_α_ atoms from MD trajectory according to the following equation

(1) $$C=\langle \left(r_{i}-\langle r_{i}\rangle \right){\left(r_{j}-\langle r_{j}\rangle \right)}^{T}\rangle \;\left(i,j=1,2,3,\cdots ,3N\right)$$

where $r_{i}$ represents the Cartesian coordinates of the $C_{\alpha }$ atom of the *i*th residue, the symbol N indicates the number of the $C_{\alpha }$ atoms of BRD4 and CBP used in the current work. The BRD4 and CBP structures extracted from MD were superimposed on the reference structure, and eliminated all translations and rotations. Then the average was computed over the structural ensembles collected from MD simulations. The covariance matrix *C* in Eq. (1) is symmetric and can be changed into a diagonal matrix *Ʌ* of eigenvalues $\lambda_{i}$ using an orthogonal transformation matrix *T*:

(2) $$\Lambda =T^{T}C_{i,j}T$$

where the columns are the eigenvectors correlating with eigenvalues, the PCs of movement can be evaluated by using the eigenvectors correlating with the first few bigger eigenvalues.

### 2.4 Residue–inhibitor interaction decomposition

In the current work, the interactions of F3J, EX1, and E2T with each residue in BRD4 and CBP were calculated by employing the MM-GBSA [15] decomposition program in AMBER18. The calculations are summarized as the following:

(3) $$\Delta G_{inhibitor-residue}=\Delta E_{vdw}+\Delta E_{ele}+\Delta G_{pol}+\Delta G_{nopol}$$

in which $\Delta E_{vdw}$, $\Delta E_{ele}$, $\Delta G_{pol}$, and $\Delta G_{nopol}$ respectively represent van der Waals interactions, electrostatic interactions of inhibitors with residues, polar contributions and non-polar contributions to inhibitor-residue interactions. All energy components were calculated by utilizing 200 snapshots taken from the last 120 ns MD trajectories with a time interval of 600 ps.

## Ethics Statement

N/A

## CRediT Author Statement

**Shiliang Wu:** Data curation, Writing - original draft, Writing - review & editing; **Lifei Wang:** Visualization, Investigation; **Lulu Zhang:** Visualization, Investigation; **Xiaoyan Xu:** Supervision; **Juan Zhao:** Validation, Writing-review & editing.

## Declaration of Competing Interest

The authors declare that they have no known competing financial interests or personal relationships which have or could be perceived to have influenced the work reported in this article.
